# Polymeric Nanoparticles in Targeted Drug Delivery: Unveiling the Impact of Polymer Characterization and Fabrication

**DOI:** 10.3390/polym17070833

**Published:** 2025-03-21

**Authors:** Lina Eltaib

**Affiliations:** Department of Pharmaceutics, College of Pharmacy, Northern Border University, Rafha, Saudi Arabia; linasalaheldin@gmail.com

**Keywords:** polymeric nanoparticles (PNPs), targeted drug delivery, polymer characterization, fabrication techniques, polymer formulations

## Abstract

Polymeric nanoparticles (PNPs) represent a groundbreaking advancement in targeted drug delivery, offering significant benefits over conventional systems. This includes their versatility, biocompatibility, and ability to encapsulate diverse therapeutic agents and provide controlled release, improving efficacy while minimizing side effects. The polymers used in PNP formulations are critical, as they influence the nanoparticles’ physicochemical properties such as size, shape, surface charge, and drug-loading capacity. Recent developments in polymer chemistry and nanotechnology have led to the creation of smart PNPs that can respond to specific stimuli, enabling precise drug release in targeted environments. This review explores the mechanisms of drug delivery, innovations in polymeric formulations, and the fabrication and characterization techniques that enhance drug delivery systems. Additionally, it discusses challenges and future directions in the field, highlighting the potential for personalized medicine and the role of artificial intelligence in optimizing nanoparticle design. By examining the relationship between polymer characteristics and PNP performance, the review aims to promote innovative therapeutic strategies in modern medicine. Despite the promise of polymer-based drug delivery systems, challenges such as toxicity, stability, scalability, and regulatory compliance must be addressed. Future research should focus on rigorous testing, clear risk communication, and sustainable practices to support clinical translation and commercial viability. Overall, the integration of these elements is crucial for advancing PNPs in therapeutic applications.

## 1. Introduction

Polymeric nanoparticles (PNPs) can encapsulate a variety of therapeutic agents, enhance bioavailability, and facilitate controlled release, thereby improving therapeutic efficacy and minimizing side effects [1,2]. The characterization of polymers used in the formulation of PNPs is crucial, as it directly influences the physicochemical properties of the nanoparticles, including size, shape, surface charge, and drug-loading capacity [3].

The effectiveness of PNPs in targeted drug delivery is largely determined by their ability to navigate biological barriers and selectively release their payload at the desired site of action. This necessitates a thorough understanding of the polymer’s characteristics, such as molecular weight, hydrophilicity/hydrophobicity, and functionalization, which can significantly affect the interaction between PNPs and biological environments [4]. Recent advancements in polymer chemistry and nanotechnology have led to the development of smart PNPs that respond to specific stimuli (e.g., pH, temperature, or enzymes) to achieve controlled drug release, thereby enhancing the therapeutic outcome [5].

Formulation methods and characterization play a pivotal role in the efficacy of nanoparticle-based drug delivery systems by influencing their physicochemical properties, which in turn affect drug delivery efficiency, targeting capabilities, and therapeutic outcomes. The characterization of nanoparticles involves assessing their size, shape, surface charge, and other properties, which are crucial for optimizing their performance in drug delivery applications. This process ensures that the nanoparticles are tailored to meet specific therapeutic needs, thereby enhancing their effectiveness in delivering drugs to target sites.

The aim of this review paper is to address the existing knowledge gap regarding the impacts of formulation and characterization on polymer properties and PNP performance. By elucidating the relationship between formulation techniques and these variables, this review seeks to pave the way for innovative therapeutic strategies.

## 2. Background

### 2.1. Polymeric Nanoparticle Dosage Forms

Polymeric nanoparticles (PNPs) represent a promising advancement in drug delivery systems, particularly in oral solid dosage forms and lipid-based formulations. Their incorporation into pellets and minitablets enhances the solubility and stability of drugs like indomethacin, facilitating more effective therapies for conditions such as inflammation and cancer [6]. Solid lipid nanoparticles (SLNs) and nanostructured lipid carriers further exemplify the potential of these systems by improving bioavailability and enabling targeted drug delivery while minimizing side effects [7]. The ability of SLNs to provide a stable, biocompatible delivery platform for poorly soluble drugs is particularly significant in the treatment of complex diseases like cancer and cardiovascular disorders [8,9,10,11]. Overall, the ongoing research and development in this field hold great promise for enhancing therapeutic efficacy and patient outcomes.

### 2.2. Overview of Polymeric Nanoparticles

Polymeric nanoparticles (PNPs) represent a significant advancement in drug delivery systems, offering enhanced therapeutic efficacy and reduced toxicity for a variety of pharmaceutical applications. The versatility of PNPs, including polymeric micelles, liposomes, and dendrimers, allows for improved compatibility with lipophilic and oil-soluble drugs, making them particularly useful for drugs with poor solubility and stability [11,12]. The incorporation of both natural and synthetic polymers, such as chitosan, poly(lactic acid), and hydroxypropyl methylcellulose, contributes to the development of biodegradable systems that support controlled drug release and stability [8,13]. Furthermore, the emergence of smart polymers, which respond to specific physiological conditions, marks a transformative approach in drug delivery. These stimuli-responsive polymers facilitate targeted and controlled release, thereby minimizing off-target effects and maximizing therapeutic outcomes [14]. The integration of such advanced materials into drug delivery systems not only enhances the efficacy of treatments but also paves the way for personalized medicine, optimizing drug utilization while reducing side effects [14]. As research continues to evolve in this field, the potential for PNPs to revolutionize drug delivery remains promising, underscoring the importance of ongoing innovation and development.

### 2.3. Mechanisms of Drug Delivery to Enhance Targeting and Efficiency

Surface modification techniques, such as PEGylation, significantly enhance the targeting efficiency of nanoparticles by minimizing interactions with non-target tissues and facilitating better penetration into target areas [15]. For example, PEGylated nanocrystals have demonstrated improved mucosal penetration and reduced mucin binding, which boosts their delivery efficiency for targeted applications [16]. Additionally, incorporating active targeting ligands like antibodies, peptides, and folates onto polymeric nanoparticles can direct therapeutic agents specifically to tumor cells, leading to increased drug accumulation at the target site while minimizing off-target effects [17].

Stimuli-responsive systems further improve drug delivery precision by enabling nanoparticles to release drugs in response to specific biological signals or environmental changes [18]. Moreover, polymeric nanoparticles exhibit high drug loading capacity, allowing for the encapsulation of various therapeutic substances. The choice of polymer and drug encapsulation method can influence the release kinetics, facilitating sustained and controlled drug release tailored to therapeutic needs [19].

Advances in polymer chemistry are also addressing biological barriers, with biodegradable and bio-based polymers promoting safer pharmaceutical practices [11]. Notably, polymeric micelles and nanoparticles can exploit the Enhanced Permeability and Retention (EPR) effect to passively target tumors while also showing promise in improving drug transport across the Blood–Brain Barrier (BBB), which is crucial for treating central nervous system disorders [20].

Recent experimental data have demonstrated the role of various nanoparticle (NP) strategies in enhancing targeting and efficiency in drug delivery and therapeutic applications. These strategies leverage the unique properties of nanoparticles to improve the precision and effectiveness of treatments, particularly in cancer therapy. The following sections detail the experimental findings from recent studies that support these claims.

### 2.4. Tandem Paired Nicking (TPN) for Genome Editing

TPN utilizes Cas9 nickases to introduce site-specific nicks on DNA, facilitating efficient homology-directed recombination for targeted knock-in. This method enhances targeting efficiency by using 1700–2000-bp donor DNAs and 20-nt-long spacers, which are crucial for precise genome editing without triggering p53-mediated DNA damage responses [21].

### 2.5. Magnetic Nanoparticles for Hyperthermia

Magnetic nanoparticles (MNPs) modified with nucleus and mitochondria-targeting peptides have shown enhanced intracellular hyperthermia efficiency. These peptides improve the uptake of MNPs in specific organelles, with mitochondria-targeting sequences demonstrating superior magnetic induction hyperthermia performance compared to nuclear localization signals [22].

### 2.6. PLGA Microspheres for Drug Delivery

Lornoxicam-loaded PLGA microspheres have been developed for intra-articular administration, showing improved targeting efficiency by reducing systemic drug leakage and increasing retention time in synovial fluid. This method prolongs drug release and enhances targeting to the site of action, minimizing systemic side effects [23].

### 2.7. HPMA Copolymer Conjugates for Tumor Penetration

HPMA copolymer conjugates of pirarubicin (P-THP) exhibit superior penetration and cytotoxicity in tumor cell spheroids. P-THP penetrates deeper into the tumor tissues and maintains cytotoxicity comparable to free drugs, despite lower cellular uptake, due to enhanced permeability and retention effects [24].

### 2.8. Dynamic Nuclear Polarization (DNP) for NMR Enhancement

DNP using non-covalent interactions between radicals and target molecules, such as BDPA and triphenylphosphine, significantly enhances 31P-NMR signals. This method demonstrates the potential of non-covalent interactions to improve signal sensitivity in NMR applications [25].

### 2.9. Co-Delivery of Drugs via Multifunctional Nanoparticles

Co-delivery of docetaxel and perifosine using Fol/R7 nanoparticles enhances anti-cancer activity by regulating the PI3K/Akt signaling pathway. These nanoparticles show increased cytotoxicity, cellular uptake, and apoptosis in drug-resistant cancer cells, highlighting their potential for overcoming drug resistance [26].

### 2.10. Targeted Nanoparticles for Overcoming Drug Resistance

Anti-P-glycoprotein conjugated nanoparticles target drug delivery to multi-drug-resistant cancer cells, enhancing cellular uptake and overcoming resistance mechanisms. This approach demonstrates the specificity and potential of targeted delivery systems in cancer treatment [27].

### 2.11. Gold Nanoparticles for Nuclear Targeting

Gold nanoparticles (GNPs) targeted to the nucleus can enhance therapeutic responses by increasing DNA damage through additional free radical production during irradiation. This strategy underscores the potential of nuclear-targeted therapies in improving cancer treatment outcomes [28].

### 2.12. Bioinspired Nano-Prodrugs for Tumor Targeting

A bioinspired nano-prodrug (BiNp) with folic-acid-targeting moieties shows significant tumor-targeting ability and enhanced uptake by cancer cells. The prodrug releases its active components in response to the acidic tumor microenvironment, promoting apoptosis and improving therapeutic efficiency [29].

### 2.13. Mesoporous Silica Nanoparticles for Drug Delivery

Mesoporous silica nanoparticles (MSNs) with high drug loading capacity and encapsulation stability are functionalized with targeting ligands for efficient cancer treatment. These MSNs demonstrate enhanced therapeutic efficiency through targeted delivery and controlled drug release [30].

While these studies highlight the advancements in nanoparticle-based targeting and efficiency, it is important to consider the challenges and limitations associated with these technologies. Factors such as biocompatibility, potential toxicity, and the complexity of manufacturing processes need to be addressed to ensure the safe and effective application of these strategies in clinical settings. Additionally, further research is required to optimize these systems for broader therapeutic applications.

### 2.14. Drug Delivery Capabilities of Polymeric Nanoparticles

Nanoemulsions made by polymeric nanoparticles are a fascinating area of research due to their potential applications in various fields such as enhanced oil recovery, antibacterial treatments, and drug delivery systems. These nanoemulsions are typically stabilized by nanoparticles, which help maintain their stability and functionality. The following (Table 1) provide examples of nanoemulsions made by nanoparticles, highlighting their composition, preparation methods, and applications.

Nanosuspensions are a promising formulation strategy for enhancing the solubility and bioavailability of poorly water-soluble drugs [36]. They consist of drug nanoparticles stabilized in a liquid medium, often using surfactants or polymers. Various examples of nanosuspensions have been developed using different methods and stabilizing agents, each tailored to the specific properties and requirements of the active pharmaceutical ingredients (APIs). Below are some notable examples of nanosuspensions made by nanoparticles, as discussed in the provided papers (Table 2).

Nanoparticles have revolutionized drug development and delivery, offering innovative solutions for various medical challenges. These tiny particles, often less than 100 nm in size, are used to enhance the solubility, bioavailability, and targeted delivery of drugs, thereby improving therapeutic outcomes. Several drugs have been developed using nanoparticle technology, particularly in the treatment of cancer and other chronic diseases. Below are examples of drugs made using nanoparticles, highlighting their applications and benefits (Table 3).

How can polymer nanoparticle manipulation improve targeted drug delivery systems?

Polymeric nanoparticles (PNPs) are gaining prominence due to their versatile applications in drug delivery, diagnostics, and material science. The fabrication techniques of these nanoparticles play a crucial role in determining their properties and functionalities. Recent advancements in fabrication methods have significantly enhanced the potential of PNPs, making them more efficient and adaptable for various applications. This answer explores the emerging role of fabrication techniques in the development of polymeric nanoparticles, highlighting key methods and their implications (Table 4).

## 3. Fabrication Techniques for Polymeric Nanoparticles

### 3.1. Polymeric Formulations Demonstrates the Best Drug Loading and Release Efficiency Performance

The evaluation of polymeric formulations for drug loading and release efficiency reveals a diverse range of materials and techniques, each with unique advantages and limitations. Among the formulations discussed, chitosan-based nanogels, mesoporous silica, and poly(ε-caprolactone) nanocapsules, as well as interpenetrating polymeric networks, demonstrate notable performance in drug delivery applications. Each formulation’s efficiency is influenced by factors such as polymer composition, crosslinking methods, and the physicochemical properties of the drug being delivered. Below is a detailed analysis of the key formulations and their performance metrics.

Nanocomposite Hydrogel Films

- **Composition and Crosslinking**: These hydrogels are formed using N-vinylpyrrolidone and sodium alginate, crosslinked with Zn^2+^ ions, and reinforced with bentonite nanoclay. This dual crosslinking strategy enhances mechanical properties and drug loading capacity [73].
- **Drug Loading and Release**: Nafcillin was loaded with an efficiency of up to 30%. The release profile was moderate, with activity against certain bacteria, which was influenced by the presence of zinc ions [74].

Chitosan-Based Nanogels

- **pH-Responsive Behavior**: These nanogels are engineered for the sustained release of madecassoside, with high swelling and drug release at pH 7.4. The formulation’s efficiency is enhanced by the chitosan content, which affects both loading and release.
- **Drug Loading and Release**: The nanogels exhibit high drug loading and controlled release, following non-Fickian diffusion kinetics, making them suitable for sustained drug delivery [75].

Mesoporous Silica and Polymeric Nanocapsules

- **Formulation Characteristics**: Ivermectin-loaded mesoporous silica (IVM-MCM) and poly(ε-caprolactone) nanocapsules (IVM-NCs) show enhanced solubility and release profiles. IVM-NCs, in particular, provide a sustained release over 72 h.
- **Drug Loading and Release**: IVM-MCM achieves a 10% *w*/*w* drug loading, while IVM-NCs achieve 0.1% *w*/*w* with 100% encapsulation efficiency, highlighting the potential for controlled release applications [76].

Polymeric Micelles and Machine Learning

- **Micelle Formation**: Poly(2-oxazoline)s and poly(2-oxazine)s form micelles that enhance the solubility of hydrophobic drugs. Machine learning models predict drug loading efficiencies, aiding in the optimization of these systems.
- **Drug Loading and Release**: These micelles demonstrate potential for high drug loading and efficient release, with models achieving balanced accuracies of 0.8 in validation tests [76]

Hydrophobic Ion Pairing (HIP)

- **Methodology**: HIP increases the lipophilicity of metformin, enhancing its loading efficiency in alginate beads. This method demonstrates a significant increase in drug loading and controlled release at specific pH levels.
- **Drug Loading and Release**: The approach results in an 88% increase in metformin loading, showcasing the effectiveness of HIP in modulating drug properties for improved delivery [77].

While each polymeric formulation offers distinct advantages, the choice of the best system depends on the specific application requirements, such as the desired release profile, drug properties, and target site. The integration of advanced techniques like machine learning for predicting drug loading efficiencies further enhances the potential of these systems in drug delivery applications. However, challenges such as scalability, biocompatibility, and regulatory approval remain critical considerations for the clinical translation of these technologies.

### 3.2. Comparative Analysis of Polymeric Nanoparticles Fabrication Methods Used in Drug Delivery

Various fabrication methods have been developed to produce PNPs, each with distinct advantages and limitations. This analysis provides a detailed comparison of the most commonly used PNP fabrication methods, focusing on their principles, advantages, disadvantages, and applications in drug delivery. The choice of fabrication method depends on the desired properties of the nanoparticles, such as size, drug loading, and release kinetics.

A comparative analysis of the key methods is presented below (Table 5).

Microfluidics-based approaches and Electrohydrodynamic (EHD) methods have emerged as powerful tools in nanoparticle synthesis and manipulation, offering several advantages over traditional techniques. EHD methods offer a wide range of advantages for nanoparticle synthesis and manipulation, including rapid processing, uniform size control, material versatility, scalability, and precise control over synthesis parameters. These methods are particularly valuable in biomedical applications and offer significant environmental and cost benefits. As research continues to advance, EHD techniques are poised to play a pivotal role in the development of next-generation nanomaterials.

These methods leverage the interaction of electric fields with charged particles in a fluid to create, shape, and assemble nanoparticles with precision. Below, we explore the key benefits of EHD methods, supported by insights from recent research papers.

Advantages of electrohydrodynamic methods for nanoparticle synthesis and manipulation (Table 6).

Advantages of Microfluidics-Based Approaches for Nanoparticle Synthesis and Manipulation.

Microfluidics-based approaches have emerged as a transformative technology in the field of nanoparticle synthesis and manipulation, offering numerous advantages over conventional methods. These benefits span precise control over nanoparticle properties, including high reproducibility, efficient production, and scalability, making microfluidics a powerful tool in advancing nanotechnology, particularly in drug delivery and biomedical research (Table 7).

### 3.3. Evaluation Methods for Polymeric Nanoparticles

Polymeric nanoparticles (PNPs) are a versatile and promising platform in nanotechnology, particularly for drug delivery and biomedical applications. Evaluating these nanoparticles involves various methods to assess their size, drug loading, stability, and interaction with biological systems. The evaluation results can vary significantly depending on the methods used, and comparing these results is crucial for optimizing nanoparticle design and application. This answer will explore the evaluation methods, results, and comparisons of polymeric nanoparticles based on the provided research papers.

#### 3.3.1. Evaluation Methods

- Dynamic Light Scattering (DLS): This technique is commonly used for measuring the size distribution of nanoparticles. It is effective for particles larger than 8 nm but may not be reliable for smaller particles due to its limitations in resolving polydisperse and non-spherical nanoparticles [104,105].
- Atomic Force Microscopy (AFM) and AFM-IR: AFM provides high-resolution imaging of nanoparticles, while AFM-IR combines AFM with infrared spectroscopy to map and evaluate drug distribution within nanoparticles. This method allows for precise quantification of drug loading and distribution at the nanoscale [106].
- Cryo-Electron Microscopy (Cryo-EM): This method is particularly reliable for analyzing small nanoparticles (<7 nm), providing detailed structural information [104]
- Nanoparticle Tracking Analysis (NTA) and Tunable Resistive Pulse Sensing (TRPS): These methods are used to measure particle size and concentration, with TRPS detecting more nanoparticles in suspension compared to NTA [105].

#### 3.3.2. Evaluation Results

- Size and Stability: The size and stability of polymeric nanoparticles can be influenced by the choice of polymer and environmental conditions. For instance, lipid-polymer nanoparticles formed from different polymers show varying stability across pH and salinity conditions [107]
- Drug Loading and Release: The AFM-IR technique has revealed significant variability in drug loading among individual nanoparticles, with loadings ranging from 0 to 21 wt%. This highlights the importance of precise characterization for quality control in nanomedicine [106]. Mathematical models such as the Tanh function and First-order model have been used to predict drug release kinetics, showing high correlations with experimental data [108].
- Protein Corona Formation: The interaction of nanoparticles with proteins in biological systems can affect their identity and fate. Some polymeric nanoparticles exhibit negligible protein corona formation, which is advantageous for maintaining their intended function in vivo [109].

#### 3.3.3. Comparison of Evaluation Methods

- Reliability and Resolution: Cryo-EM is more reliable for small nanoparticles, while DLS is suitable for larger particles but may not resolve polydispersity effectively. Combining multiple techniques, such as AF4-MALS-UV-DLS, can provide a comprehensive characterization of nanoparticle size and distribution [104,105].
- Drug Distribution Analysis: AFM-IR offers a unique advantage in mapping drug distribution within nanoparticles, which is not possible with traditional methods like DLS or NTA [106]
- Environmental Stability: The stability of nanoparticles under different conditions can be assessed using absorbance and DLS measurements, with some polymers showing greater stability across a range of pH and salinity levels [107].

While the evaluation methods for polymeric nanoparticles provide valuable insights into their properties and performance, each method has its limitations and strengths. The choice of evaluation technique can significantly impact the interpretation of results, and combining multiple methods can offer a more comprehensive understanding. Additionally, the development of new techniques, such as AFM-IR, enhances the ability to precisely characterize drug loading and distribution, which is crucial for the advancement of nanomedicine.

Experimental examples for the impact of physiochemical properties

The physicochemical properties of polymers, such as molecular weight, charge, and degradation rates, are critical in determining their performance and applications. Experimental studies have provided insights into how these properties change under various conditions, influencing polymer stability and functionality. This answer synthesizes findings from several studies to illustrate experimental examples of these properties.

#### 3.3.4. Molecular Weight

- Measurement Techniques: Gel permeation chromatography (GPC) is a common method used to monitor changes in molecular weight during polymer degradation. For instance, studies on polystyrene (PS) and polypropylene (PP) exposed to UV irradiation showed that degradation rates are fastest near the exposed surface, with molecular weight decreasing significantly due to photooxidation [110].
- Degradation Dynamics: The molecular weight of polymers like polystyrene can significantly affect degradation dynamics. Experiments have shown that polystyrene degrades primarily through random-chain scission, with the rate of degradation being influenced by the initial molecular weight of the polymer [111].
- Environmental Influence: In poly(DL-lactide-co-glycolide) (PLGA) films, molecular weight decreases were observed under accelerated storage conditions, with changes in humidity and temperature affecting the rate of degradation [112].

#### 3.3.5. Charge

- Charge Effects on Degradation: While the provided papers do not directly address the charge of polymers, it is known that the presence of charged groups can influence polymer interactions with the environment, potentially affecting degradation rates. For example, charged groups can enhance water uptake, which may accelerate hydrolytic degradation in certain polymers.

#### 3.3.6. Degradation Rates

- Experimental Devices: Devices designed to study polymer degradation, such as those involving UV irradiation and controlled aqueous environments, help in understanding how various factors like light and pH influence degradation rates [113].
- Thermal and Mechanical Factors: The degradation of polymers such as isotactic polypropylene (iPP) involves thermo-oxidative processes, where oxygen diffusion into the amorphous phase leads to chain scission and molecular weight reduction [114] Similarly, high-pressure conditions in GPC can cause mechanical degradation, as observed in polystyrene samples [115].
- Chemical and Radiative Influences: The degradation of polymers can also be influenced by chemical and radiative factors, which alter the molecular weight distribution and lead to changes in physical properties such as color and crystallinity [116].

While the focus here is on molecular weight and degradation rates, it is important to consider that polymer charge; though not extensively covered in the provided papers, it plays a crucial role in applications involving electrostatic interactions and conductivity. Additionally, the interplay between molecular weight, charge, and degradation rates can be complex, with each factor potentially influencing the others in various environmental and experimental contexts. Understanding these interactions is essential for designing polymers with tailored properties for specific applications.

The integration of artificial intelligence (AI) in nanoparticle design:

Artificial intelligence holds significant promise for advancing personalized medicine and cancer treatment. AI-assisted nanoparticle design can enhance the precision and efficacy of drug delivery systems, optimize therapeutic outcomes, and minimize side effects. By leveraging AI, researchers can tailor nanoparticles to individual patient profiles, improving the targeting and effectiveness of cancer therapies. This approach not only enhances the therapeutic index but also paves the way for more personalized and efficient cancer treatments. Below are the key applications and benefits of AI-assisted nanoparticle design in personalized medicine and cancer treatment.

### 3.4. Enhanced Drug Delivery Systems

- An AI-driven design of nanoparticle-based drug delivery systems allows for meticulous drug delivery, improved bioavailability, and reduced side effects. AI methods such as machine learning and neural networks facilitate the creation of nanoparticles with tailored characteristics, including size, surface chemistry, and drug release profiles, which are crucial for targeted therapy [117].
- AI can optimize nanocarrier design to ensure precise and controlled drug release, enhancing therapeutic benefits while minimizing adverse effects. This is particularly beneficial in cancer treatment, where targeted delivery can significantly improve patient outcomes [118,119].

### 3.5. Personalized Treatment Strategies

- AI analyzes patient data, including clinical and genetic information, to predict outcomes and recommend personalized treatment plans. This enables the development of nanoparticles that are specifically designed to meet the therapeutic needs of individual patients, thus enhancing the precision of cancer treatments [117,120].
- In liver cancer, for example, AI-powered algorithms optimize nanocarrier design and facilitate real-time monitoring of treatment efficacy, allowing for more accurate patient stratification and treatment personalization [121].

### 3.6. Improved Diagnostic and Predictive Capabilities

- AI enhances diagnostic accuracy and predictive modeling, which are essential for developing personalized treatment strategies. By integrating AI with nanotechnology, clinicians can achieve more precise patient stratification and improve clinical decision-making, ultimately leading to better patient outcomes [121,122].
- AI’s ability to analyze large datasets and predict nanoparticle behavior in biological environments is crucial for enhancing targeting specificity and reducing off-target effects, which are common challenges in cancer therapy [116,123].

### 3.7. Overcoming Challenges in Cancer Treatment

- AI-assisted nanoparticle design can address challenges such as drug resistance and tumor heterogeneity by enabling the development of nanoparticles that bypass efflux pumps and target cancer stem cells. This is vital for overcoming multidrug resistance and improving the efficacy of cancer therapies [118].
- The integration of AI in nanomedicine also facilitates the development of “smart” nanoparticles that respond to environmental triggers, further enhancing the precision and effectiveness of cancer treatments [118].
- While AI-assisted nanoparticle design offers numerous benefits, there are challenges and considerations that must be addressed. Issues such as data integration, algorithm transparency, and regulatory hurdles pose significant challenges to the widespread adoption of AI in nanomedicine. Additionally, ethical concerns and the need for large-scale clinical validation remain critical barriers to the full realization of AI’s potential in personalized medicine and cancer treatment [119,124,125]. Addressing these challenges will be essential to harness the full potential of AI-assisted nanoparticle design in transforming cancer therapy.

## 4. Characterization of Polymeric Nanoparticles

- Characterization of polymer nanoparticles is crucial in drug formulation to ensure their efficacy, stability, and safety. These nanoparticles, due to their small size and high surface area, require precise characterization techniques to evaluate their physical and chemical properties. Various methods are employed to analyze aspects such as size, morphology, surface charge, chemical composition, and drug loading capacity. The integration of these techniques provides a comprehensive understanding of polymer nanoparticles, which is essential for their application in drug delivery systems.
- The characterization of polymer nanoparticles is critical for advancing their applications in drug delivery systems. Various techniques, including Transmission Electron Microscopy (TEM), Scanning Electron Microscopy (SEM), and Atomic Force Microscopy (AFM), provide insights into the size, morphology, and surface characteristics of these nanoparticles. Spectroscopic methods such as Fourier Transform Infrared Spectroscopy (FTIR) and Nuclear Magnetic Resonance (NMR) Spectroscopy further elucidate the chemical composition and molecular structure, while techniques like Dynamic Light Scattering (DLS) and X-ray Diffraction (XRD) help in assessing size distribution and crystalline structure [126,127,128]. Despite the advancements in characterization techniques, challenges such as polydispersity and stability of nanoparticles persist. The integration of multiple characterization methods can enhance the reliability of results and provide a more comprehensive understanding of nanoparticle behavior. This multi-modal approach not only addresses the limitations of individual techniques but also aids in the development of improved drug delivery systems. Furthermore, considerations regarding biocompatibility and adherence to regulatory standards are essential for the successful transition of these nanoparticles from laboratory research to clinical applications [83,129].

### 4.1. Surface Characterization

**Figure 1:**
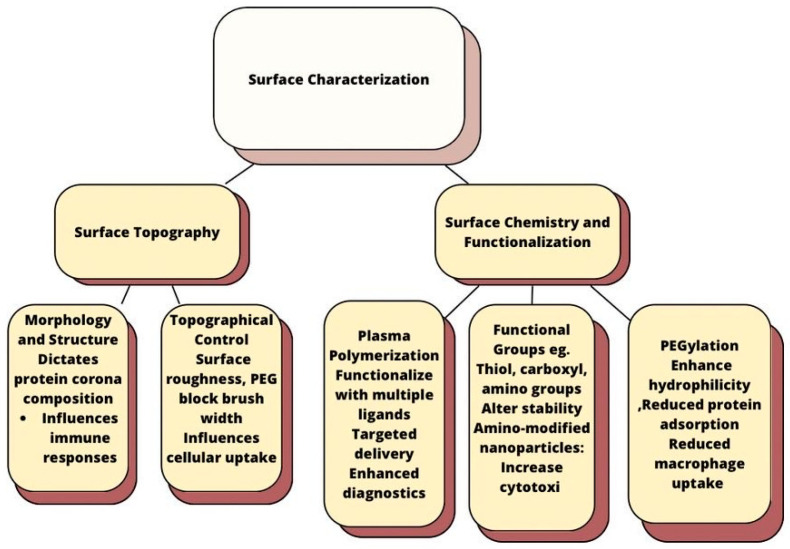
Surface characterization.

- Its understanding is useful to modify nanoparticle surfaces to enhance their potential for targeted delivery and diagnostics without significantly altering their physicochemical properties. It is summarized as follows (Figure 1):

### 4.2. Integration of Nanotechnology with Biologics

The integration of nanotechnology with biologics presents significant advancements, particularly in the realm of protein corona formation and cellular uptake. The formation of a protein corona on nanoparticle surfaces is crucial as it defines the biological identity of these nanoparticles, influencing their recognition and processing by the immune system, which in turn affects their biodistribution and clearance [130]. Furthermore, surface modifications of nanoparticles can play a pivotal role in enhancing or hindering cellular uptake. For instance, engineering nanoparticles with flexible surfaces can improve their adhesion and uptake by adjusting stiffness and membrane interactions [131]. Additionally, the incorporation of polyethylene glycol (PEG) and amine groups can significantly modulate interactions with biological barriers, such as mucus and the extracellular matrix, thereby facilitating or impeding transport [132]. Collectively, these factors highlight the importance of understanding the interplay between nanotechnology and biological systems to optimize the design of nanoparticle-based therapeutics.

### 4.3. The Role of Characterization in the Development of Nanoparticle-Based Drug Delivery Systems

Polymer characterization techniques such as Nuclear Magnetic Resonance (NMR), Fourier Transform Infrared Spectroscopy (FTIR), and Differential Scanning Calorimetry (DSC) play a crucial role in influencing drug release rates and stability in drug delivery systems. These techniques provide insights into the physicochemical properties of polymers, which are essential for designing effective drug delivery systems. By understanding the interactions between drugs and polymers, researchers can optimize drug loading, release rates, and stability, ultimately enhancing therapeutic efficacy and patient outcomes. The following sections detail the importance of each technique in this context.

While these techniques are invaluable for optimizing drug delivery systems, challenges remain, such as the need for more advanced methods to accurately determine drug-polymer miscibility at room temperature [133]. Additionally, the potential toxicity and stability issues of polymer-based systems highlight the importance of continued research and development to address these limitations and improve drug delivery efficiency [134].

#### 4.3.1. Importance of Nanoparticle Size and Shape

The size and shape of nanoparticles play a crucial role in their effectiveness for drug delivery and therapeutic applications. Smaller nanoparticles are particularly advantageous as they can easily cross biological barriers, such as the blood--brain barrier, making them suitable for targeting the central nervous system, which is essential for treating cognitive defects [78,79]. Additionally, the shape of the nanoparticles influences their cellular uptake and distribution; for example, cylindrical or needle-like nanoparticles may enhance cellular uptake compared to their spherical counterparts, thus offering potential benefits in directing drug delivery [79].

#### 4.3.2. Surface Charge and Functionalization

The surface charge of nanoparticles, indicated by their zeta potential, significantly influences their interaction with cell membranes, with positively charged nanoparticles generally exhibiting better absorption by negatively charged cells, thereby enhancing targeting efficiency [79]. Additionally, functionalization through modifications such as coating with biocompatible materials or ligand conjugation can further improve the uptake of nanoparticles by target cells. For instance, chitosan-coated nanoparticles have demonstrated enhanced solubility and controlled drug release in pH-sensitive environments [80].

#### 4.3.3. Physicochemical Characterization Techniques

Analytical methods such as Transmission Electron Microscopy (TEM), Scanning Electron Microscopy (SEM), and Raman Spectroscopy are utilized to characterize the size, shape, and surface properties of nanoparticles, providing valuable insights into the stability, reproducibility, and efficacy of nanoparticle formulations [135,136]. Additionally, non-destructive testing techniques like Dynamic Light Scattering and X-ray Diffraction are employed to assess the physicochemical parameters of nanocarriers, which are essential for understanding their physiological performance [137].

#### 4.3.4. Impact on Drug Release and Stability

Characterization plays a crucial role in the design of nanoparticles for drug delivery, particularly in achieving controlled release and ensuring stability. By developing nanoparticles with specific release profiles, such as pH-sensitive formulations that respond to the acidic environment of tumor tissues, drugs can be delivered precisely when and where they are needed, thereby enhancing therapeutic efficacy [138]. Additionally, proper characterization is essential for maintaining the stability of these nanoparticles, preventing premature degradation and ensuring consistent drug delivery over time [139].

### 4.4. Challenges and Advancements in Nanoparticle Characterization Methodologies

Characterizing nanoparticles is a crucial aspect of nanotechnology, as it allows for the exploration and enhancement of their unique properties for various applications. The methodologies for nanoparticle characterization have evolved significantly, offering detailed insights into their size, shape, surface characteristics, and chemical composition. However, these methodologies also face several challenges, including the need for high precision, reproducibility, and the ability to handle complex matrices. This answer explores the challenges and advancements in nanoparticle characterization methodologies, drawing from a range of techniques and their applications.

#### 4.4.1. Complexity and Diversity of Nanoparticles

Nanoparticles present a diverse array of characteristics, including variations in size, shape, composition, and surface properties, which complicates the development of a universal characterization method. Each characterization technique possesses its own strengths and limitations, making it challenging to select the most appropriate method for specific nanoparticles. Proper sample preparation is essential to prevent agglomeration, as this can skew results; maintaining the dispersion of nanoparticles in a manner that reflects their natural state poses a significant challenge. Furthermore, achieving reproducible results across different laboratories and studies is hindered by variations in techniques and equipment, highlighting the urgent need for standardization in nanoparticle characterization to ensure consistency and reliability.

#### 4.4.2. Challenges in Polymer-Based Drug Delivery Systems

Polymer-based drug delivery systems face several challenges, including potential toxicity, stability issues, and manufacturing complexities, which necessitate ongoing research to address these limitations. Additionally, developing innovative manufacturing techniques and establishing clearer regulatory pathways are crucial for their clinical application [134,140]. The complexity of nanoparticle formulation further complicates large-scale production and regulatory approval, requiring rigorous characterization to ensure consistency and safety [141]. Moreover, the subcellular size of nanoparticles raises concerns about potential adverse effects, while the long-term impacts on human health and the environment remain significant issues [142].

### 4.5. Considerations for Clinical Translation

Addressing immunogenicity, stability under physiological conditions, and production scalability is essential for the clinical use of nanoparticle-based drugs [54]. While artificial intelligence (AI) can optimize formulations, it raises concerns about prediction reliability and overfitting, which may result in suboptimal drug designs. Additionally, the development and production of these advanced drug delivery systems may be more expensive than traditional formulations, potentially limiting their widespread adoption and accessibility in the healthcare market.

### 4.6. Advancements and Potential Solutions

The integration of nanotechnology and smart polymers holds promise for more efficient, patient-centric therapies, as highlighted by [69,134]. By combining nanotechnology with personalized medicine, treatment specificity can be significantly enhanced, as noted by [143]. Additionally, optimizing drug delivery systems through innovative polymer formulations not only improves therapeutic outcomes but also ensures patient safety and environmental sustainability.

### 4.7. Future Prospects and Directions in Research

Despite the advancements in PNP fabrication, several challenges remain, including scalability, reproducibility, and biocompatibility. Future research should focus on developing multi-functional nanoparticles with stimuli-responsive properties for targeted drug delivery [144,145]. Additionally, the integration of machine learning and artificial intelligence in nanoparticle design could revolutionize the field by optimizing fabrication parameters and predicting nanoparticle behavior [146,147].

The integration of polymeric nanoparticles with personalized medicine approaches holds the potential to create highly effective, patient-specific treatments, leading to significant improvements in therapeutic outcomes for various diseases. Future research is likely to focus on overcoming current challenges such as scalability, reproducibility, and long-term safety of these nanoparticles, as ensuring consistent quality and performance in large-scale production is crucial for their widespread adoption. Additionally, beyond oncology, polymeric nanoparticles are being explored for applications in treating cardiovascular, neurological, and infectious diseases, indicating their broad applicability in modern medicine.

## 5. Conclusions

- Polymeric nanoparticles offer immense potential in drug delivery due to their versatility, biocompatibility, and ability to provide controlled release profiles. The choice of fabrication method depends on the specific requirements of the drug and the desired properties of the nanoparticles. Continued advancements in fabrication techniques and materials science will further enhance the applications of PNPs in medicine. This comprehensive analysis highlights the strengths and limitations of various PNP fabrication methods, providing a foundation for selecting the most appropriate technique for specific drug delivery applications.
- Polymer-based drug delivery systems offer transformative potential, but overcoming challenges related to toxicity, stability, scalability, and regulation is crucial.
- Future research should focus on rigorous testing, transparent risk communication, and sustainable practices to facilitate clinical translation and commercial success.
- Characterization plays a fundamental role in the development of nanoparticle-based drug delivery systems by ensuring their physicochemical properties align with therapeutic requirements, thereby enhancing drug stability, targeting efficiency, and controlled release. Techniques such as electron microscopy, zeta potential analysis, and functionalization strategies allow for the precise tailoring of nanoparticles to optimize their performance in clinical applications. Despite advancements, challenges such as reproducibility, scalability, and regulatory hurdles remain significant barriers to widespread adoption. Future research must focus on overcoming these limitations through standardized characterization methodologies, innovative polymer formulations, and integration with personalized medicine approaches. The continued evolution of nanoparticle characterization techniques, coupled with advancements in nanotechnology and artificial intelligence, will pave the way for more effective, patient-specific drug delivery systems, ultimately improving treatment outcomes across various medical fields.

## Figures and Tables

**Table 1:** Examples of nanoemulsions, highlighting their stabilizing agent, key features, and applications.

Nanoemulsion	Stabilizing Agent	Key Features	Applications
Anionic Surfactant-Alumina Nanoparticle Stabilized Nanoemulsion [31]	Alumina Nanoparticles and Sodium Lauryl Sulfate	Synthesized using high-energy ultrasound; robust stability and viscoelastic properties; stability due to electrostatic repulsion; a balance of solid and elastic properties.	Enhanced Oil Recovery (EOR)
Cationic Metal Nanoparticle-Conjugated FusogenicNanoemulsion (CFusoN) [32]	Cationic Metal Nanoparticles	Achieves 99.999% killing efficiency against Staphylococcus aureus; causes membrane depolarization and lipid solubilization; no hemolytic activity or cytotoxicity.	Antibacterial applications
Starch Nanoparticle Stabilized Pickering Nanoemulsion [33]	Starch Nanoparticles	Prepared using ultrasonication and high-pressure homogenization; used for carotenoid-enriched powders; significant reduction in the particle size enhances stability and bioavailability.	Food and Nutraceuticals
Resveratrol Nanoemulsion [34]	Nanoparticles	Contains resveratrol for increased bioavailability; prepared with organic solvent and medium-chain fatty acid triglycerides; enhances solubility and stability for therapeutic use.	Therapeutic applications
Repaglinide Nanoemulsion [35]	Surfactants (Tween 80, Pluronic F68)	Developed to improve oral bioavailability of repaglinide; droplet sizes less than 120 nm; aims to overcome low water solubility and hepatic first-pass metabolism.	Type 2 Diabetes Treatment

**Table 2:** Examples of nanosuspension products made by nanoparticles, highlighting the technology used, key features, and purpose/indication.

Nanosuspension Product	Technology Used	Key Features	Purpose/Indication
Abraxane^®^ (nab™ paclitaxel) [36]	Nanoparticle albumin-bound (nab™)	Stabilizes drug nanoparticles; eliminates the need for solubilizers.	Cancer treatment
Fyarro^®^ (nab™ rapamycin) [36]	Nanoparticle albumin-bound (nab™)	Stabilizes drug nanoparticles; eliminates the need for solubilizers.	Treatment of certain tumors
Itraconazole Nanosuspension [36]	nab™ technology	Enhanced solubility through high-pressure homogenization.	Antifungal treatment
Albendazole Nanosuspension [37]	Nanoprecipitation with ultrasonication	Improved solubility and dissolution rates with polymers.	Anthelmintic treatment
Annonaceous Acetogenin Nanosuspension [38]	Amphiphilic stabilizers	High drug loading capacity; stability in gastrointestinal fluid.	Antineoplastic treatment
Acyclovir Nanosuspension [39]	Media milling and homogenization	Enhanced solubility and bioavailability for various routes.	Antiviral treatment
Nanosuspension Formulation of Diosmin [40]	Probe sonication with varying concentrations of different surfactants and polymers	Enhanced oral delivery.	A vascular protector for the treatment of hemorrhoids and venous leg ulcers

**Table 3:** Examples of medicinal products made by nanoparticles for specific disease areas, highlighting the formulation type, key features, and key features and benefits.

Disease Area	Drug/Product	Formulation Type	Key Features and Benefits
Cancer Treatment [41,42]	Doxil	Liposomal formulation of doxorubicin	Reduces cardiotoxicity and enhances delivery to tumor cells via the EPR effect.
Cancer Treatment [43,44]	Abraxane	Nanoparticle albumin-bound paclitaxel	Improves solubility and facilitates delivery to the tumor site.
Cardiovascular and Autoimmune Disorders [45,46]	Nanoparticle-based formulations	Various nanoparticle types (dendrimers, micelles)	Improves pharmacokinetic profiles; better targeting; reduced side effects.
Infectious Diseases [43]	Protein nanoparticles	Protein-based nanoparticles (albumin, gelatin)	Biocompatibility and biodegradability for effective drug delivery.
Neurodegenerative Diseases [45]	Nanogels and nanodiamonds	Nanogel and nanodiamond formulations	Potential to cross the blood–brain barrier for treating Alzheimer’s and Parkinson’s.
Cancer Therapy [47]	Polymeric nanoparticles	Polymeric nanoparticles	They can be engineered to respond to specific stimuli, such as pH changes in the tumor microenvironment, to release drugs at the desired location.
Multifunctional Platforms: [48]	Polymeric nanoparticles	Polymeric nanoparticles	These nanoparticles serve as theranostic platforms, combining therapeutic and diagnostic functions. They can be modified to include imaging agents, allowing for simultaneous drug delivery and monitoring of treatment efficacy

**Table 4:** Organization of the capabilities of polymer-based nanoparticle systems in drug delivery, including key features, advantages, and references.

Key Feature	Advantages	Ref.
Controlled and Sustained Release	Achieved using polymers like PLGA, PLA, and chitosan, which encapsulate drugs and enhance their release rates, targeting specific sites for improved therapeutic outcomes.	[49,50,51,52]
Biocompatibility and Biodegradability	Reduces the risk of toxicity and adverse reactions, making polymers suitable for long-term use in drug delivery systems.	[50,51]
Targeted Delivery	Functionalized with ligands like antibodies and peptides to target specific cells/tissues, enhancing drug concentration at the target site and minimizing systemic exposure.	[53,54]
Versatility in Drug Encapsulation	Can encapsulate a wide range of therapeutic agents, including small molecules, proteins, and nucleic acids; adaptable for various diseases.	[51,54]
Polymer–Drug (Conjugates–Ligand) Conjugation	Extends circulation times, enables targeted delivery, and reduces immunogenicity, particularly in oncology. Functionalization of nanoparticles with targeting ligands, such as VNAR ligands	[55,56]
Mucoadhesive Systems	Polymers with mucoadhesive properties enable localized drug delivery on mucosal surfaces (e.g., buccal, sublingual, nasal).	[57]
Stimuli-Responsive Polymers	Responds to environmental stimuli (pH, temperature, ionic strength) for targeted drug delivery, especially in ocular and nose-to-brain systems.	[57]
Membrane Technology	Nanoparticle-embedded polymers help in membrane separation processes, enhancing selectivity and permeability.	[58]
Biomedical Applications	PNPs extend to diagnostics and imaging, with techniques like chemical labeling enabling targeted delivery to cells, such as cancer cells.	[59]
Cancer Therapy: Active and Passive Targeting	Targeted delivery of chemotherapeutic agents directly to tumor sites, minimizing damage to healthy tissues and improving therapeutic outcomes. An enhanced permeability and retention (EPR) effect, allowing for the accumulation of nanoparticles in tumor tissues.	[53,60,61,62]
Overcoming Biological Barriers	Nanoscale size and surface modifications enable the penetration of biological barriers (e.g., the blood–brain barrier), facilitating drug delivery to inaccessible sites.	[54,63]
Enhanced Bioavailability	Nanoparticles increase drug dissolution rates and solubility, improving bioavailability, particularly for poorly soluble drugs.	[64,65,66,67]
Personalized Medicine	Integration with nanotechnology allows for more tailored therapeutic approaches, improving treatment efficiency.	[68,69,70,71]
Artificial Intelligence (AI) Integration	AI can analyze biological data and predict polymer interactions, enabling the design of more precise formulations tailored to patient needs.	[72]

**Table 5:** Comparative Analysis of Polymeric Nanoparticles Fabrication Methods Used in Drug Delivery.

No. and Method	Principle	Advantages	Disadvantages	Applications	Ref.
1. Methods Involving Preformed Polymers					
1.1. Nanoprecipitation	Polymer–drug mixture precipitates in a solvent upon the addition of a non-solvent.	Simple, versatile, scalable, and precise control over particle size.	Limited encapsulation efficiency for hydrophilic drugs.	Hydrophobic drugs and natural polymers.	[78]
1.2. Emulsification–Solvent Evaporation	Polymer and drug are dissolved in an organic solvent and emulsified in water, followed by solvent evaporation.	High drug encapsulation efficiency; controlled particle size.	High-energy input and the use of toxic organic solvents.	Encapsulation of hydrophobic drugs.	[79]
1.3. Emulsification–Solvent Diffusion	Uses a partially miscible solvent system to induce nanoprecipitation.	Suitable for thermosensitive drugs; rapid nanoparticle formation.	Limited control over particle size distribution.	Drugs requiring mild fabrication conditions.	[80]
1.4. Salting-Out	A high salt concentration in an aqueous phase induces nanoprecipitation of the polymer–drug mixture.	Avoids organic solvents and is environmentally friendly.	Larger particle sizes and broader size distribution.	Water-soluble polymers and drugs.	[81]
1.5. Microfluidics	Uses microfluidic devices to control mixing of the polymer, drug, and solvent streams.	Excellent control over particle size and uniformity; scalable.	Requires specialized equipment and expertise.	Tailored nanoparticles for targeted drug delivery.	[82]
2. Methods Involving Polymerization of Monomers					
2.1. Emulsion Polymerization	Monomers polymerized in an aqueous phase with surfactants.	High drug loading and encapsulation efficiency.	Requires strict control to avoid coagulation.	Synthetic polymers like polystyrene and poly(acrylic acid).	[83]
2.1. Emulsion Polymerization	Monomers polymerized in an aqueous phase with surfactants.	High drug loading and encapsulation efficiency.	Requires strict control to avoid coagulation.	Synthetic polymers like polystyrene and poly(acrylic acid).	[83]
2.2. Mini-Emulsion Polymerization	Uses mini-emulsions to stabilize monomer droplets during polymerization.	Encapsulates both hydrophobic and hydrophilic drugs.	Complex process requiring precise emulsification control.	Dual-drug delivery systems.	[84]
2.3. Micro-Emulsion Polymerization	Uses micro-emulsions to form nanoparticles.	High stability and uniform particle size.	Limited scalability due to high surfactant concentration.	Biodegradable polymers like PLGA.	[85]
3. Specialized Fabrication Methods					
3.1. Electrospinning	Uses electrostatic forces to fabricate nanofibers from polymer solutions.	High surface area, porous structure, and controlled drug release.	Limited control over fiber diameter and potential needle clogging.	Transdermal drug delivery and wound healing.	[86]
3.2. Electrospraying	Uses electrostatic forces to produce nanoparticles instead of fibers.	High throughput and uniform particle size.	Requires precise electrostatic control.	Pulmonary drug delivery and vaccine development.	[87]
3.3. Single-Chain Polymer Nanoparticles (SCPNs)	Intramolecular cross-linking of single polymer chains.	Well-defined size, high stability, and tunable functionality.	Complex synthesis requiring specialized monomers.	Nanomedicine and imaging.	[54]
3.4. Phase Separation-Induced Nanoprecipitation	Phase separation in a miscible solvent–water system co-precipitates the polymer and drug.	High drug loading (up to 66.5 wt%) and high encapsulation efficiency (>90%).	Limited to specific solvent systems.	Poorly soluble drugs.	[88]

**Table 6:** Advantages of EHD methods for nanoparticle synthesis.

Advantage	Description	Ref.
Rapid Synthesis	Synthesis in minutes or milliseconds.	[89,90]
Uniform Size and Shape Control	Production of monodisperse nanoparticles.	[91,92]
Versatility in Material Synthesis	Suitable for proteins, polymers, metals, and high-entropy alloys.	[89,90,93]
Scalability and Efficiency	Scalable methods with reduced material consumption.	[90,92]
Precise Control over Parameters	Control over electric fields, mixing, and real-time monitoring.	[89,94]
Enhanced Material Properties	High surface areas, catalytic activity, and structural integrity.	[89,95]
Manipulation and Assembly	Precise control over nanoparticle position, size, and assembly into nanosheets.	[90,92]
Biomedical Applications	Production of biocompatible, biodegradable nanoparticles for drug delivery.	[89,96]
Environmental and Cost Benefits	Minimal material waste and reduced experimental costs.	[90,92]
Integration with Other Techniques	Combination with plasma synthesis, stochastic electrochemistry, and more.	[90,97]

**Table 7:** Advantages of microfluidics-based approaches for nanoparticle synthesis and manipulation.

Advantage	Description	Ref.
Precise Control over Properties	Enables precise control over size, morphology, and size distribution of the nanoparticles.	[98,99,100]
High Reproducibility	Ensures consistent production with minimal batch-to-batch variability.	[98,99]
Continuous and Efficient Production	Allows for steady and uninterrupted production, enhancing efficiency.	[100,101]
Scalability	Facilitates large-scale production, addressing translational challenges.	[100,101]
Functionalization	Enables modification of the nanoparticles for specific applications.	[102,103]

## Abbreviations

The following abbreviations are used in this manuscript:

PNPs	polymeric nanoparticles
